# Construction and Validation of Academic Support Scale in Middle School (ASSMS)

**DOI:** 10.3390/bs14110981

**Published:** 2024-10-22

**Authors:** Zeqing Zheng, Wujun Sun, Ping Fang, Lina Chen

**Affiliations:** 1 State Key Laboratory of Cognitive Neuroscience and Learning, Beijing Normal University, Beijing 100875, China; 2 Faculty of Education, Henan Normal University, Xinxiang 453007, China; 3 School of Psychology, Capital Normal University, Beijing 100048, China; 4 College of Education, Hengshui University, Hengshui 053000, China

**Keywords:** academic support, middle school students, development

## Abstract

Research on social support in China has been relatively comprehensive as psychological development cannot be separated from the social environment. Family, teachers, and friends are the main sources of social support for middle school students. If we can fully utilize the academic support provided by families, teachers, and classmates in daily life, improve students’ personal efficacy, stimulate their learning motivation and desire, and provide corresponding emotional, resource, behavioral, and willingness support for middle school students, it will help them better enhance their learning adaptability, increase their investment in learning, and then improve their learning consciousness, ultimately promoting the enhancement of academic achievement and the development of their abilities. Therefore, based on the existing scales of social support and academic support, this study determined the academic support scale for middle school students, exploring the source of academic support from four dimensions: willingness support, resource support, emotional support, and behavioral support. Based on a literature review and questionnaire, a formal scale was formed to measure the academic support of middle school students. In total, 762 students from two middle schools in Shijiazhuang and Hengshui were selected as subjects for data analysis. Exploratory factor analysis showed that the scale was composed of four factors: willingness support, resource support, emotional support, and behavioral support, with a cumulative contribution rate of 54.15%. The factor loadings of the items ranged from 0.423 to 0.783. The indicators obtained by confirmatory factor analysis were χ^2^/df of 2.98, RMSEA of 0.072, GFI of 0.841, IFI of 0.902, TLI of 0.891, and CFI of 0.901. The Cronbach’s α coefficient was 0.955, the Spearman–Brown coefficient was 0.939, and the correlation coefficients between each dimension and the total scale were 0.892–0.945. The results show that the reliability and validity of the scale are within the acceptable range, in line with the standards of measurement, and can be used as a tool to measure the academic support of middle school students. This measurement can be used to evaluate the multidimensional academic support systems in middle schools and inform the development of targeted interventions to promote student success and well-being.

## 1. Introduction

Middle school is a critical transition period in a student’s educational journey, marked by significant academic, social, and emotional changes [1]. Adequate academic support during this time can significantly impact student success and well-being [2]. Academic support helps students navigate the challenges of middle school, such as developing study habits for the new educational environment and navigating more mature interpersonal relationships with family members, teachers, and classmates [3,4] and the need for greater independence [5].

Based on a review of existing research on the effects of academic support from different sources, the key points concern the structure of existing measurement tools. Studies have explored the positive effects of support from different sources, including parents, teachers, and peers, on students’ academic outcomes [6,7]. The literature highlights the important role of teacher support, teacher–student relationships, and the quality of teaching in influencing students’ engagement, workload perception, and learning outcomes [8,9,10]. Peer support and social relationships among students also play a crucial role in predicting academic performance and persistence, especially during the transition to higher education [11,12,13]. Family support, particularly emotional support, has been linked to various developmental and academic outcomes, such as adjustment to college, academic and social development, and academic performance [14,15,16,17,18]. Also, the existing research suggests that academic support encompasses various dimensions, including emotional support, informational support, evaluative support, and cognitive support [19]. These dimensions of support can be provided by different sources, such as parents, teachers, and peers, and can have distinct effects on students’ academic outcomes.

However, existing measures of academic support may have limitations in terms of breadth, specificity, or psychometric properties [20,21]. Many existing scales measure social support more broadly, without a specific focus on academic support. For example, the Multidimensional Scale of Perceived Social Support (MSPSS) assesses individuals’ perceptions of social support from friends, family, and significant others, but does not differentiate between general social support and academic-specific support [22]. Some scales, such as the Inventory of Socially Supportive Behaviors (ISSB), focus on the reception of support from an individual’s broader social network, without addressing the unique needs and challenges faced by middle school students in the academic domain [23]. Scales like the Student Academic Support Scale (SASS) have been developed and validated with college student populations, but their psychometric properties may not generalize well to the middle school context [24,25]. Similarly, the Child and Adolescent Social Support Scale (CASSS), while designed for children and adolescents, may require further validation to ensure its reliability and validity for assessing academic support in middle school students, especially in diverse cultural contexts [19].

The existing research on the value of social and academic support measures has some limitations and inconsistencies. While the academic support scales are often derived from broader social support measures, they may not adequately capture the specific needs and challenges faced by middle school students in the academic domain. Additionally, the subjects of some scales, such as ISSB and SASS, are college students, rather than middle school students. The CASSS is closer to the concept and target population of academic support for middle school students, but its reliability and validity may need further verification, especially in the context of research extended to China. Therefore, the development of a scale specifically designed to measure the multidimensional nature of academic support for middle school students, with strong psychometric properties, could provide a valuable tool for researchers and educators to better understand and address the academic support needs of this population. Furthermore, new assessment tools can fill gaps in existing tools and meet diverse needs, as existing assessment tools may fail to take into account the diversity of different student groups or the effectiveness of specific forms of support.

As is shown in the above-mentioned literature, it is necessary to consider the development of students from the aspects of teachers, family, and friends. However, this definition across these three aspects is not enough. Support has a great influence on middle school students’ study and life. What kind of support do they need? The two types of support include subjective and objective support in learning from surroundings. Subjective support refers to an individual’s subjective perception of others’ support or the availability of others’ support when necessary. Support for learning includes small groups, individualized teaching methods, teachers who care, and practical and emotional support [26]. Moreover, perceived emotional support has been found to provide a buffering effect against the stressful experience of being alone in a new environment for first-year university undergraduate students [27]. Emotional and information support were also found to be the most frequently reported social support types, with information support reported by many previous authors [19,28,29]. Moreover, we should also consider whether the people around us are willing to provide students with learning help. Therefore, this study compiles the subjective content of academic support from the perspective of emotional support and willingness support.

Another objective social support refers to the actual support behavior that an individual receives, especially in the process of an online course [30]. In recent years, it has been suggested that a student should be given tips, recommendations, and guidance about his/her learning behaviors using learning analytics [31,32]. Previous research found that appraisal support, which is also called advice or cognitive guidance, helps individuals define, understand, and cope with problematic events by providing evaluation and feedback [33]. Instrumental support defines students’ problem solving by providing tangible help or information when needed [34], which has a positive relationship with students [19]. However, material support and instrumental support provided by others have received little attention in the academic context [27]. We focus on the help of specific behaviors that students feel they can provide in the learning process and resource support that others can provide.

Finally, we developed a questionnaire suitable for measuring the academic support of middle school students based on an existing questionnaire, considering the literature on sources and dimensions and experts’ suggestions. The questionnaire is expected to have four dimensions, including willing support, resource support, emotional support, and behavior support. In this paper, by modifying the questionnaire and collecting the data, an effective and reliable tool is developed after analysis.

## 2. Methods

### 2.1. Research Framework

Based on an extensive review of the existing literature, this study established a comprehensive research framework consisting of four key dimensions of academic support: willingness support, resource support, emotional support, and behavioral support. This multidimensional conceptualization was designed to thoroughly probe the complex phenomenon of academic support among middle school students. In the scale development process, the research team referred to well-established measures such as MSPSS, ISSB, SASS, and CASSS. The aim was to compile informants that closely align with the language habits and understanding abilities of the target middle school student population. Scale development involved a rigorous review process, with 25 psychologists (20 master’s students, 4 doctoral students, and a professor) providing expert input. The research team carefully examined the compiled items, eliminating any redundant or inconsistent questions and refining the wording to ensure clarity and logical coherence.

The final academic support scale encompasses 4 dimensions and 41 items, with a 5-point Likert-type response format ranging from “very inconsistent” to “very consistent”. This comprehensive measurement tool provides a robust framework to capture the multifaceted nature of academic support experienced by middle school students.

### 2.2. Participants

The data for this study were collected from 762 middle school students in Shijiazhuang and Hengshui, Hebei Province. Three classes were randomly selected ranging from junior one to senior two, and all students and their parents provided informed consent. A total of 800 paper questionnaires were distributed, and 762 were collected, resulting in a recovery rate of 95.25%. The sample consisted of 385 female (50.5%) and 370 male (48.6%) students, with 7 students (0.9%) not providing gender information. The grade distribution was as follows: junior one (21.1%), junior two (21.0%), junior three (19.3%), senior one (19.2%), and senior two (19.4%). After item analysis with all valid data, the research team identified experience-based content from two data sources, and 50% of the samples were randomly selected for exploratory factor analysis (EFA) and confirmatory factor analysis (CFA).

All procedures were approved by Hengshui University. The methods were conducted following relevant guidelines and regulations, adhering to the Declaration of Helsinki for the protection of human research participants. All participants and their parents volunteered and provided informed consent.

### 2.3. Measurement and Instruments

To ensure a smooth data collection process, the researchers first established contact with the participating middle schools, introducing the research content and procedures to the principals and teachers. The questionnaires were then distributed and collected by the teachers on-site. Each dimension and the total score of the scales were calculated using the imputed scores. To mitigate potential bias, the researchers emphasized the confidentiality of responses and encouraged honest self-reflection. Additionally, the use of a paper questionnaire rather than a digital tool was based on the context of the study, and the paper format helped ensure that all participants had equal access to the material. The data were analyzed and processed using SPSS 24.0 and Amos 24.0 software. The research team carefully screened the questionnaire based on the results of the data analysis, ensuring the reliability and validity of the final academic support scale.

## 3. Results

### 3.1. Item Analysis

All data were analyzed using SPSS 24.0. The main methods included item analysis, high-low group comparison, and homogeneity testing. First, 762 valid responses were processed to address any missing values or extreme values. Since there were no reverse-coded items in the initial questionnaire, no special handling was required. The 41 items in the questionnaire were then analyzed. The total scores of all participants were calculated, and an independent sample t-test was conducted. The total scores were ranked from high to low, and the scores of the top and bottom 27% of participants were identified as the high and low groups, respectively. An independent sample t-test was used to test the difference between the high and low groups for each item. All the results were significant, with a critical ratio (CR) greater than 3.00. Next, a homogeneity test was carried out in which the correlation coefficients of all items and total scores were examined. Items with an overall scale correlation coefficient of less than 0.4 were considered for deletion. All the items met this criterion. Finally, a comprehensive evaluation of the indicators mentioned above was conducted. All the items were found to meet the required standards and were included in the subsequent exploratory factor analysis.

### 3.2. Exploratory Factor Analysis (EFA)

There were 762 valid responses, and 50% of the samples were randomly selected for exploratory factor analysis and confirmatory factor analysis. After the item analysis, exploratory factor analysis was carried out for all the retained items. As shown in Table 1, the items of the scale are suitable for factor analysis. The KMO value is 0.939, which is very suitable for factor analysis according to the standard. Additionally, the chi-square approximation of Bartlett’s spherical test is 5390.977 (*p* < 0.001), indicating that the sample data are very suitable for factor analysis.

The principal component analysis was used to extract common factors, the correlation matrix was analyzed, and the orthogonal rotation method was used to rotate factors. According to the results of factor rotation, the items were screened again according to the following criteria: ① the highest load value is less than 0.40, ② the factor commonality is less than 0.45, and ③ the number of items corresponding to the factor is less than 3. For the items that did not meet the criteria, the items from the lowest load were deleted, and the remaining items were re-analyzed using factor analysis. According to the screening criteria, 14 items, namely V25, V6, V2, V3, V5, V7, V13, V16, V20, V28, v34, V35, V36, and V37, were deleted. Finally, 27 items were kept on the academic support scale. The specific factor structure and factor loadings are presented in Table 2.

**Figure 1:**
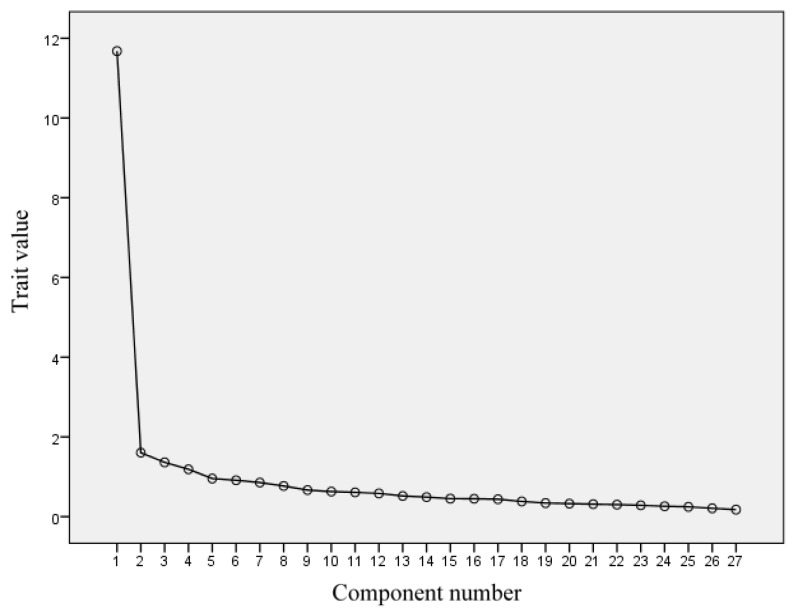
Gravel map.

According to the analysis results and the scree plot, four factors were selected. The cumulative contribution rate of variance was 58.641%, as shown in Table 3 and Figure 1.

Based on the analysis results and the theoretical foundation, the items corresponding to each factor were named:

Factor 1: “Willing Support”—This factor mainly reflects the willingness or ability of teachers, family members, and classmates/friends to provide academic support. It includes 8 items.

Factor 2: “Resource Support”—This factor primarily reflects the conditions and assistance provided by teachers, family members, and classmates/friends to support learning. It includes 6 items.

Factor 3: “Emotional Support”—This factor mainly reflects the emotional recognition and support from teachers, family members, and classmates in the learning process. It contains 8 items.

Factor 4: “Behavioral Support”—This factor refers to the specific behavioral help provided by teachers, family, and classmates to support learning, such as answering questions. It contains 5 items.

### 3.3. Confirmatory Factor Analysis (CFA) About Academic Support

The remaining half of the data were used for confirmatory factor analysis. The model’s goodness of fit is reflected by the fit indices shown in Table 4. The goodness of fit index x^2^/df value is less than 3.00, the root mean square error (RMSEA) is between 0.05 and 0.08, the GFI is greater than 0.80, and the TLI, IFI, and CFI are close to 0.90. These results indicate that the fit indices of this model are acceptable, and the overall model fit is good. This suggests that the scale has good structural validity.

### 3.4. Reliability Test and Validity of Academic Support Scale

In this study, Cronbach’s α coefficient was used as the internal consistency reliability index, and the Spearman–Brown coefficient was used as the split-half reliability index. The results show that Cronbach’s α coefficient and the split-half reliability of the total scale are 0.955 and 0.939, respectively. At the same time, Cronbach’s α coefficient of each dimension is 0.872, 0.823, 0.865, and 0.770, and the Spearman-Brown coefficient of each dimension is 0.822, 0.838, 0.855, and 0.751. All of these values are above 0.75, indicating that the scale has high reliability.

Construct validity refers to the extent to which a test measures the theoretical structure or psychological characteristics that the researcher wants to measure. In addition to confirmatory factor analysis, the correlation between the factors and the total scale was analyzed to further test the structural validity of the scale. The test results are shown in Table 5. According to the results, there is a significant positive correlation between each dimension of the scale and the total scale, with correlation coefficients ranging from 0.892 to 0.945. There is also a high correlation between each dimension, with correlation coefficients between 0.775 and 0.847. These findings indicate that the scale has good structural validity.

## 4. Discussion and Conclusions

Academic support is a comprehensive concept with rich connotations, involving multiple levels and contents, not only considering family, teachers, and peers but also psychological and non-psychological factors. This study proposes and validates a more comprehensive and holistic measurement framework to reflect the construct of academic support for middle school students.

Firstly, the scale measures both subjective and objective support, capturing both the psychological and non-psychological aspects of academic support. Psychological support refers to emotional encouragement and cognitive guidance provided by significant others, while non-psychological support encompasses instrumental and material assistance [34,35]. In this study, willingness support and emotional support belong to the psychological support dimension, while resource support and behavioral support fall under the non-psychological support dimension. Integrating these two types of support within a unified framework and measurement tool helps to better understand the multifaceted nature of academic support.

Secondly, the scale assesses academic support from various sources, including family, teachers, and peers. This approach reflects the comprehensive nature of academic support that students receive from their surrounding environment. Evaluating support from different sources is beneficial as it allows for a more nuanced understanding of the differential impacts of family, teacher, and peer support on students’ academic development [6,13,36].

The four-dimensional structure of this academic support scale, encompassing emotional support, willingness support, behavioral support, and resource support, demonstrates its ability to comprehensively interpret the content of academic support for middle school students. This multidimensional conceptualization is consistent with the theoretical model of social support, which distinguishes between action-facilitating support and nurturant support [36].

Empirical evidence suggests that these dimensions of academic support are related to various positive outcomes for students, such as mental health, academic adjustment, and confidence in academic work [27,37,38]. Forming supportive relationships through academic support has also been identified as an important outcome of the student academic support process [38]

In summary, the academic support scale developed in this study provides a comprehensive and psychometrically sound measurement tool that captures the multidimensional nature of academic support for middle school students. By considering both subjective and objective support, as well as support from different sources, this scale offers a robust framework for understanding and addressing the academic support needs of this population. It is helpful to monitor the longitudinal impact of support systems on student outcomes and facilitate collaboration between educators, families, and community stakeholders to holistically meet students’ needs.

## 5. Limitations and Future Directions

Despite the strengths of this academic support measure, the current study has several limitations that warrant consideration in future research. First, the sample size, while large enough for the current analysis, could be expanded to further enhance the generalizability of the findings. Second, the study primarily relied on internal consistency reliability and structural validity, and additional validation analyses, such as test–retest reliability and criterion-related validity, would strengthen the psychometric properties of the scale.

Furthermore, the current scale does not differentiate the relative importance or frequency of various support behaviors from different sources. Incorporating such information could improve the clinical interpretability of the scale [25]. Additionally, collecting data from multiple informants, including teachers, parents, and peers, would provide a more comprehensive understanding of students’ academic support experiences and increase the reliability of the scale.

Finally, the applicability of this scale is currently limited to middle school students in China. Examining the cultural and contextual generalizability of the scale in other countries and educational settings would be a valuable future direction. It might also be beneficial to explore, in future research, the possibility that academic support might vary based on factors such as socioeconomic status or geographic location.

Overall, this study offers a robust and comprehensive measure of academic support for middle school students, serving as a valuable tool for researchers and practitioners to better understand and address the academic support needs of this population. The limitations identified provide opportunities for future research to further refine and expand the utility of this academic support measurement framework.

## Figures and Tables

**Table 1:** KMO and Bartlett test.

KMO		0.930
Bartlett test	chi-square approximate value	5390.977
	df	351
	Sig.	0.000

**Table 2:** Factor structure and load value of Academic Support Scale.

	1	2	3	4
V39	0.723			
V29	0.723			
V11	0.704			
V38	0.699			
V14	0.660			
V17	0.577			
V27	0.556			
V15	0.540			
V23		0.707		
V30		0.699		
V9		0.637		
V24		0.607		
V31		0.550		
V26		0.517		
V19		0.441		
V22		0.423		
V41			0.723	
V18			0.696	
V21			0.681	
V32			0.653	
V12			0.548	
V10			0.489	
V8				0.783
V1				0.711
V4				0.659
V33				0.534
V40				0.489

**Table 3:** Characteristic values and variance contribution rates of three factors.

Component	Eigenvalue	Variance Percentage	Accumulation%
1	4.733	17.528	17.528
2	4.019	14.884	32.412
3	3.900	14.445	46.857
4	3.182	11.784	58.641

**Table 4:** Fit index of confirmatory factor analysis.

x^2^	df	x^2^/df	RMSEA	GFI	TLI	IFI	CFI
948.545	318	2.983	0.072	0.841	0.891	0.902	0.901

**Table 5:** Correlation coefficients of the academic support scale and its subscales.

	Willing Support	Resource Support	Emotion Support	Behavior Support	Total Scale
Willing support	1				
Resource support	0.828 **	1			
Emotion support	0.845 **	0.847 **	1		
Behavior support	0.800 **	0.781 **	0.775 **	1	
Total scale	0.945 **	0.933 **	0.944 **	0.892 **	1

** *p* < 0.01.

## Data Availability

The data that support the findings of this study are available from the corresponding author upon reasonable request.

## References

[1] Eccles J.S., Roeser R.W. (2011). Schools as developmental contexts during adolescence. J. Res. Adolesc..

[2] Schwerdt G., West M.R. (2013). The impact of alternative grade configurations on student outcomes through middle and high school. J. Public Econ..

[3] Estévez I., Rodríguez-Llorente C., Piñeiro I., González-Suárez R., Valle A. (2021). School engagement, academic achievement, and self-regulated learning. Sustainability.

[4] Credé M., Niehorster S. (2012). Adjustment to college as measured by the student adaptation to college questionnaire: A quantitative review of its structure and relationships with correlates and consequences. Educ. Psychol. Rev..

[5] Wang M., Eccles J.S. (2012). Adolescent behavioral, emotional, and cognitive engagement trajectories in school and their differential relations to educational success. J. Res. Adolesc..

[6] Alfaro E.C., Umaña-Taylor A.J., Bámaca M.Y. (2006). The influence of academic support on Latino adolescents’ academic motivation. Fam. Relat..

[7] Ganotice F.A., King R.B. (2014). Social influences on students’ academic engagement and science achievement. Psychol. Stud..

[8] Jackling B., De Lange P., Phillips J., Sewell J. (2012). Attitudes towards accounting: Differences between Australian and international students. Account. Res. J..

[9] Lipka O., Forkosh Baruch A., Meer Y. (2019). Academic support model for post-secondary school students with learning disabilities: Student and instructor perceptions. Int. J. Incl. Educ..

[10] Nyadanu S.D., Garglo M.Y., Adampah T., Garglo R.L. (2015). The impact of lecturer-student relationship on self-esteem and academic performance at higher education. J. Soc. Sci. Stud..

[11] Altermatt E.R. (2019). Academic support from peers as a predictor of academic self-efficacy among college students. J. Coll. Stud. Retent. Res. Theory Pract..

[12] Dennis J.M., Phinney J.S., Chuateco L.I. (2005). The role of motivation, parental support, and peer support in the academic success of ethnic minority first-generation college students. J. Coll. Stud. Dev..

[13] Gallop C.J., Bastien N. (2016). Supporting Success: Aboriginal Students in Higher Education. Can. J. High. Educ..

[14] DeBerard M.S., Spielmans G.I., Julka D.L. (2004). Predictors of academic achievement and retention among college freshmen: A longitudinal study. Coll. Stud. J..

[15] Guiffrida D.A. (2004). Friends from home: Asset and liability to African American students attending a predominantly White institution. J. Stud. Aff. Res. Pract..

[16] Roksa J., Kinsley P. (2019). The role of family support in facilitating academic success of low-income students. Res. High. Educ..

[17] Scott L., Harper S., Boggan M. (2012). Promotion of Arts Integration to build Social and Academic Development. Natl. Teach. Educ. J..

[18] Wintre M.G., Yaffe M. (2000). First-year students’ adjustment to university life as a function of relationships with parents. J. Adolesc. Res..

[19] Malecki C.K., Demaray M.K. (2003). What type of support do they need? Investigating student adjustment as related to emotional, informational, appraisal, and instrumental support. Sch. Psychol. Q..

[20] Rosenfeld L.B., Richman J.M., Bowen G.L. (2000). Social support networks and school outcomes: The centrality of the teacher. Child Adolesc. Soc. Work J..

[21] Suldo S.M., Gelley C.D., Roth R.A., Bateman L.P. (2015). Influence of peer social experiences on positive and negative indicators of mental health among high school students. Psychol. Sch..

[22] Zimet G.D., Dahlem N.W., Zimet S.G., Farley G.K. (1988). The multidimensional scale of perceived social support. J. Personal. Assess..

[23] Barrera Jr M., Sandler I.N., Ramsay T.B. (1981). Preliminary development of a scale of social support: Studies on college students. Am. J. Community Psychol..

[24] Thompson B., Mazer J.P. (2009). College student ratings of student academic support: Frequency, importance, and modes of communication. Commun. Educ..

[25] Thompson B., Mazer J.P. (2012). Development of the parental academic support scale: Frequency, importance, and modes of communication. Commun. Educ..

[26] Bolic Baric V., Hellberg K., Kjellberg A., Hemmingsson H. (2016). Support for learning goes beyond academic support: Voices of students with Asperger’s disorder and attention deficit hyperactivity disorder. Autism.

[27] Wilcox P., Winn S., Fyvie-Gauld M. (2005). ‘It was nothing to do with the university, it was just the people’: The role of social support in the first-year experience of higher education. Stud. High. Educ..

[28] Chernyshenko O.S., Kankaraš M., Drasgow F. (2018). Social and emotional skills for student success and well-being: Conceptual framework for the OECD study on social and emotional skills. OECD Education Working Papers.

[29] Frydenberg E., Martin A.J., Collie R.J. (2017). Social and emotional learning in Australia and the Asia-Pacific. Social and Emotional Learning in the Australasian Context.

[30] Van Wyk M.M. (2021). Academic support under COVID-19 lockdown: What students think of online support e-tools in an ODeL course. Interact. Technol. Smart Educ..

[31] Jivet I., Scheffel M., Specht M., Drachsler H. License to evaluate: Preparing learning analytics dashboards for educational practice. Proceedings of the 8th International Conference on Learning Analytics and Knowledge.

[32] Karaoglan Yilmaz F.G. (2020). Modeling Different Variables in Flipped Classrooms Supported with Learning Analytics Feedback= Ögrenme Analitigi Geribildirimleri ile Desteklenmis Ters-Yüz Ögrenme Ortaminin Çesitli Degiskenler Açisindan Modellenmesi. Online Submiss..

[33] Brown G.W., Andrews B. (1986). Social support and depression. Dynamics of Stress: Physiological, Psychological and Social Perspectives.

[34] Semmer N.K., Elfering A., Jacobshagen N., Perrot T., Beehr T.A., Boos N. (2008). The emotional meaning of instrumental social support. Int. J. Stress Manag..

[35] Haley W.E., Levine E.G., Brown S.L., Bartolucci A.A. (1987). Stress, appraisal, coping, and social support as predictors of adaptational outcome among dementia caregivers. Psychol. Aging.

[36] Cutrona C.E., Suhr J.A. (1992). Controllability of stressful events and satisfaction with spouse support behaviors. Commun. Res..

[37] Whiteman S.D., McHale S.M., Crouter A.C. (2011). Family relationships from adolescence to early adulthood: Changes in the family system following firstborns’ leaving home. J. Res. Adolesc..

[38] Thompson R.A. (2008). Early attachment and later development: Familiar questions, new answers. Handbook of Attachment: Theory, Research, and Clinical Applications.

